# Community-Based Diabetes Screening and Risk Assessment in Rural West Virginia

**DOI:** 10.1155/2016/2456518

**Published:** 2016-01-12

**Authors:** Ranjita Misra, Cindy Fitch, David Roberts, Dana Wright

**Affiliations:** ^1^Department of Social & Behavioral Sciences, Robert C Byrd Health Science Center, School of Public Health, West Virginia University, 3313A, Morgantown, WV 26506-9190, USA; ^2^Programs and Research, Extension Service, West Virginia University, P.O. Box 6031, 812 Knapp Hall, Morgantown, WV 26506-6031, USA; ^3^WVU Extension Service, Lincoln and Boone Counties Extension Agent, Hamlin, WV, USA; ^4^WVU Extension Services, 815 Alderson Street, Williamson, WV 25661, USA

## Abstract

This project utilized a cross-sectional study design to assess diabetes risk among 540 individuals from 12 counties using trained extension agents and community organizations in West Virginia. Individuals were screened for diabetes using (1) the validated 7-item diabetes risk assessment survey and (2) hemoglobin A1c tests. Demographic and lifestyle behaviors were also collected. The average age, body mass index, and A1c were 51.2 ± 16.4, 31.1 ± 7.5, and 5.8 ± 0.74, respectively. The majority were females, Non-Hispanic Whites with no prior diagnosis of diabetes. Screenings showed that 61.8% of participants were at high risk for diabetes. Family history of diabetes (siblings or parents), overweight or obese status, sedentary lifestyle, and older age were commonly prevalent risk factors. Higher risk scores computed from the 7-item questions correlated positively with higher A1c (*r* = 0.221, *P* < 0.001). In multivariate logistic regression analyses, higher diabetes risk was predicted by obesity, older age, family history of hypertension, and gestational diabetes. Females were 4 times at higher risk than males. The findings indicated that community-based screenings were an effective way to assess diabetes risk in rural West Virginia. Linking diabetes screenings with referrals to lifestyle programs for high risk individuals can help reduce the burden of diabetes in the state.

## 1. Introduction

Diabetes affects 29.1 million Americans [1]. In adults, type 2 diabetes mellitus (T2DM) accounts for 90 to 95% of all cases. Despite emphasis on lifestyle modification and medications to treat T2DM, half of T2DM patients remain poorly controlled [2]. Consequently, it is the 7th leading cause of death due to complications such as heart disease and stroke, kidney failure, lower-limb amputations, and new cases of blindness. The economic burden of diabetes in 2012 was $245 billion for direct medical cost and $69 billion in indirect costs, such as disability, time lost from work, and premature death [3].

*West Virginia Ranks 2nd in Prevalence of Diabetes among the 53 States and Territories*. According to the 2012 Behavioral Risk Factor Surveillance System (BRFSS), diabetes prevalence in West Virginia was 13% (268,000 individuals), significantly higher than the national average of 10.2% [4]. Furthermore, another study estimated that 99,800 have undiagnosed diabetes [5] and possibly complications [6]. In addition, approximately 465,000 individuals have prediabetes. Factors that contribute to high rates of diabetes and prediabetes in West Virginia include aging population, physical inactivity, obesity, geography, lack of access to quality care, and the Appalachian culture of distrust of the healthcare system. It is estimated that the number of West Virginians living with diabetes (diagnosed and undiagnosed) will increase to 17% by 2025 from 268,000 to 314,000 [7]. The resulting medical and societal cost of diabetes will be $3.0 billion, a 25% increase from 2010 [7]. The average medical expenditures for patients with diabetes (nationally) were 2.3 times higher than those without diabetes [8]; glucose control was correlated with the medical cost, increasing significantly for every one percent increase in glycosylated hemoglobin (A1C) above 7%. Diagnosis and education to improve diabetes self-care management and health outcomes are limited due to lack of the patient's awareness of diabetes and its complications and convenient local screening and prevention programs. Patient care in the rural WV southern counties is limited, and interactions of individuals with diabetes with their providers are infrequent and ineffective for outpatient visits [9, 10].

Recent estimates claim that many individuals are not fully self-aware of their risk for diabetes. Misperceptions and lack of knowledge of their actual risk can put them on a fast track to developing diabetes if they do not reduce risk factors such as being overweight or obese, smoking, and physical inactivity. For example, in an international survey released by Health Dialog, 74% of American respondents said that obesity, unhealthy diet, and inadequate physical activity levels constitute the nation's biggest health issues [11]. Yet, 51% of the American respondents that were obese considered themselves to be healthy and 43% thought that their diets were good. This disconnect between what they know and how they apply that knowledge to themselves is quite concerning. Diabetes and its resulting comorbidities are of grave concern. Furthermore, evidence that behavioral lifestyle interventions can prevent or delay the development of type 2 diabetes and reduce risk factors for cardiovascular disease has been demonstrated in the US Diabetes Prevention Program [12, 13]. However, identification of individuals who are undiagnosed cases or at high risk is paramount before any intervention program can be launched to improve diabetes and CVD risk factors in community settings [14–16]. Several recent translations of the Diabetes Prevention Program and Finnish Diabetes Prevention Study have demonstrated encouraging effects across diverse settings, including churches [17, 18], community settings [16, 19], and YMCAs [18]. The personnel who implemented the intervention included health care professionals like nurses but also volunteer medical personnel, YMCA trainers, and community health workers.

Hence, the purpose if this study was to assess individual's diabetes risk in twelve rural counties of West Virginia using a noninvasive survey, followed up with glycosylated hemoglobin or A1c test to identify individuals with prediabetes and higher risk for diabetes. All high risk cases were referred to a self-management education and support program, the Dinning with Diabetes & Diabetes Prevention Program, and encouraged to visit a healthcare provider for follow-up care.

## 2. Methodology

The current project utilized a cross-sectional study design to assess diabetes risk using a partnership between the academic community, extension agents, and community organizations. The screenings were completed by extension agents who were part of our investigative team. Extension agents were trained to complete diabetes screenings (both surveys and A1c). They work with their local communities and provide a range of community-based educational programs including the Dining with Diabetes program. Hence, using the WVUES network to test the effectiveness of diabetes screening in W.Va. counties was innovative and would allow for long-term sustainability of this model.

We used an established community-based approach, a network of community organizations, and the West Virginia University Extension Service (WVUES) Network for diabetes screenings and referral using extension agents. Prior to data collection, the extension agents were involved in the planning of the project, that is, finalization of sites and community events in all the twelve counties where data collection was completed, that is, McDowell, Logan, Boone, Lincoln, Cabell, Wirt, Braxton, Clay, Wood, Berkley, Jackson, and Greenbrier counties. Based on our initial discussion with the community coalitions and community leaders, several screenings were offered during week days and weekends to maximize participation.

The Community Health Care Centers in all the twelve counties surveyed for this project offered annual health fairs that allowed community agencies to set up informational booths to showcase services available in the area. County extension agents set up booths at these community health fairs to promote their services as well as diabetes screenings. Advertisement for screenings included free A1c testing during the event. While the health fair targets the general population, the majority of individuals who came to the booth were adults with some level of interest for assessing their diabetes risk. Interested participants completed a 2-page survey focused on family and personal health history and health behaviors, followed by A1c tests at the site (A1c tests were not mandatory but encouraged).

Diabetes screenings were also offered in conjunction with other scheduled community events such as parent/teacher nights at local high schools, information booths at basketball games, and scheduled library community events. These events were targeted to capture a diverse group of individuals. For example, the local library offers regular educational opportunities on topics that range from adult literacy to financial planning. Furthermore, in rural West Virginia communities, school and local sporting events are considered an important forum of civic engagement and provided our extension agents a greater access to individuals in the communities.

Participants included 540 individuals from 12 rural counties (521 zip codes) who were assessed for their risk for diabetes. Individuals aged 18 years and older were screened since younger participants are less likely to have diabetes. There were no exclusions by gender, but pregnant women were excluded due to their conditions. The majority of individuals who stopped by the booths and tables were interested and completed the health screening survey. While it is difficult to determine the number of uninterested participants in community events such as health fairs (as they could easily avoid the booth) or other events, it is likely that individuals who came to the extension booths for diabetes screenings are more likely to have greater health consciousness and/or worried about getting diabetes. Also, diabetes screenings scheduled at community events allowed for interested individuals to have fewer distractions of free give away materials that occurred during health fairs in other booths.

Approximately 50% of participants declined to complete the diabetes screenings at the health fairs and 20% at the library and community events. Why community events provided a greater response to the diabetes screenings remains an open empirical question for further investigation, and use of various community forums allowed for a greater representative sample in our study. However, the sample was biased towards a higher educated participant group as more than half of the participants were college graduates or had some college level education.

The survey and A1c testing were administered by the trained research team and information was collected during the 12 months of the study enrollment. The primary outcome was risk for diabetes.

### 2.1. Diabetes Risk Assessment

The main focus of this project was to identify individuals at high risk for diabetes without imposing tests that are difficult to perform or sustain in a community setting. Hence, we used an approach that combines a questionnaire and point-of-care capillary glycosylated hemoglobin tests to predict the risk for prediabetes or undiagnosed diabetes [20]. This approach involved three steps. (1) Completion of a validated and reliable 7-item diabetes risk assessment survey developed by the Centers for Disease Control and Prevention (CDC; http://www.cdc.gov/diabetes/prevention/pdf/prediabetestest.pdf): the questionnaire included age, weight for height, exercise, diabetes in the family, and delivery of a large baby. (2) Collection of a drop of whole blood by finger stick to assess the glycosylated hemoglobin (A1c) using point-of-care A1c Now monitoring kit by Bayer: the use of A1c was preferred over fasting or random glucose as it requires only a drop of capillary blood (finger prick), can be drawn by the extension agents, and can be completed at any time of the day. Hence, it was appropriate for community settings and reflects the average blood glucose levels in the past 3 months. The A1c Now is available for over-the-counter or professional use and is approved by the Food and Drug Administration for monitoring of A1c [21, 22]. Sicard and Taylor have shown that A1c Now has good accuracy and high correlation with standardized laboratory testing [23]. (3) A risk score of ≥9: medical history of the participants allowed us to identify those individuals with no prior diagnosis of diabetes by a health care provider; A1c levels of 5.7 and higher with no prior diagnosis allowed to identify individuals with prediabetes or possibly undiagnosed diabetes.

### 2.2. Healthy Lifestyle Habits

We assessed healthy lifestyle behaviors using one question on smoking status “do you smoke” with response options yes or no; food label reading behavior “do you read food labels” with response options yes or no; and two questions on physical activity “how often do you exercise for periods of at least 30 minutes” and “how do you rate your overall level of physical activity.” Response options included less than once a week, 1-2 times per week, and 3-4 times per week and low, moderate, and high, respectively. Due to high correlation between the two physical activity questions, the latter was used for analysis. In addition, information was collected on participants' demographic characteristics (age, gender, educational level, self-reported height and weight so that we can calculate the BMI, and educational level).

All analyses were done using the Statistical Program for Social Sciences (SPSS) system (version 21.0). Basic descriptive statistics were obtained for demographic, lifestyle, and diabetes risk factors. ANOVA with Post Hoc analysis was used to evaluate the difference in risk score by gender and educational status of respondents. The acceptance level for statistical significance was 0.05. Logistic regression analysis was used to estimate the factors that influence diabetes risk. The dependent variable was the low risk and higher risk group of participants. The following variables were included in the model: age, gender, education, body mass index, physical activity, tobacco use, current health status, family history of chronic diseases (diabetes and hypertension), and A1c. Individuals with prior diagnosis of diabetes (*n* = 81; 15%) were excluded from the multivariate analysis.

## 3. Results and Discussion

The sample comprised 538 individuals from 12 rural counties. The average age of the participants was 51.2 ± 16.4 years (range 18–89 years). The majority was female (78.8%), Non-Hispanic White (88.3%), with no prior diagnosis of diabetes (83.9%). Approximately half of the participants (43.3%) had a high school degree or less.

In terms of lifestyle behavior, 81.9% of participants indicated they do not smoke (4.3% did not provide the information); females had slightly higher smoking rates than males, but it did not significantly vary by gender. Approximately one-third or 37.5% reported sedentary lifestyle that is, at least 30 minutes of exercise less than once a week. While it is encouraging that two-thirds of the participants indicated they read food labels, 35.4% did not. Furthermore, females were significantly more likely to read food labels (68%) as compared to males (52%) (*P* = 0.002).

Mean body mass index (BMI) was 31, in the obese category; males (32.5 ± 7.7) were significantly more obese than females (30.7 ± 7.4; *P* = 0.029). Approximately one-third of the participants or 37.4% had high blood pressure and two-thirds (67.8%) indicated they had family history of high blood pressure.

Descriptive participant characteristics for low risk and high risk for prediabetes, and prior diagnosed diabetes are presented in Table 1. Significant differences were noted between the groups by age, body mass index, family history, and some of the lifestyle behaviors. Individuals with prior diagnosis of diabetes were more likely to be older, obese, and with a family history or medical history of hypertension. In addition, they also were less likely to be smokers, do vigorous activity, and be with no prior diagnosis of diabetes. Similarly, individuals at high risk for diabetes were older individuals (45 years of age and older), were obese, had a family/medical history of hypertension, and were female. Furthermore, they were more likely to read food labels and less likely to smoke than individuals with lower risk of diabetes (Table 1).

### 3.1. Diabetes Risk

81 individuals (15.1%) indicated they had a prior diagnosis of diabetes by a healthcare professional and hence were excluded from the diabetes risk assessment. The majority of participants (61.8%) were at high risk for prediabetes as they had a risk score of 9 points or higher. Mean risk score was 9.0 ± 4.83 for all participants without prior diagnosis of diabetes; mean score was 4.15 ± 2.46 for low risk and 12.35 ± 2.78 for high risk participants. According to the CDC's risk calculator, low risk for having diabetes is 3 to 8 points and high risk is 9 or more points; those with high risk are recommended to follow-up with their health care provider. The Diabetes Risk Calculator was developed and validated using data from NHANES III [20]. Analysis of the individual seven risk factors showed that the majority of participants had a family history of diabetes (siblings or parents; 65%), weighed more than their normal weight for height (66.7%), lead a sedentary lifestyle (37%), and were older in age (>45 years; 64%), all of which increased their risk for diabetes (Table 2).

Mean A1c was 5.6 ± 0.41 for the low risk group, 5.9 ± 0.85 for high risk group, and 7.2 ± 1.8 for participants with prior diagnosis of diabetes; A1c positively correlated with higher diabetes risk scores (*r* = 0.221, *P* < 0.001).

Results from the bivariate and multivariable logistic regression analyses are presented in Table 3. Logistic regression analysis estimated the factors that influenced diabetes risk for the two groups of participants: low risk and higher risk for diabetes. Important known risk factors were controlled in the model: age, gender, education, body mass index, physical activity, tobacco use, current health status, family history of chronic diseases (diabetes and hypertension), and A1c. The results between the unadjusted and adjusted ORs were consistent, with the exception of gender; gender was statistically significant in the unadjusted models but approached significance in the adjusted models (*P* = 0.07). Association of diabetes risk score and family history of diabetes (sibling and parents), smoking status, and reading food labels were significant in the unadjusted (bivariate) model but not in the adjusted model (Table 3). As shown in the adjusted model, overweight/obese and older respondents were significantly at higher risk. However, for females, having a history of gestational diabetes increased their risk twelvefold as compared to those with low risk (OR 12.2; 95% CI 2.6–56.9). Furthermore, participants with higher risk were approximately four times as likely to have a family history of hypertension as compared to those with low risk (OR 4.08; 95% CI 1.2–14.2). Individuals with increased risk were handed their risk score and available healthcare providers in the area; they were encouraged to make an appointment with their primary care provider or a health care provider.

The burden of diabetes appears to be particularly high in rural areas of the state, with more than 60% of participants without a previous diagnosis of diabetes being at high risk of developing type 2 diabetes. This concurs with the high burden of reported diabetes and its serious health and economic consequences for the individual and society and is a major public health problem, especially in a rural, medically underserved state such as West Virginia. Over the past several years, WV has ranked among the highest in diabetes occurrence in the country [24] suggesting that West Virginians have a vulnerability that may have genetic and/or lifestyle causative links. Lack of access as well as having to travel far for care are reported as factors associated with lack of preventive healthcare, besides poor dietary habits, sedentary lifestyle, obesity, and overweight status and low health literacy which may play a role in the higher rates of diabetes in the state [25–28].

Use of community-based screenings may be critical for primary and secondary prevention, and to educate and identify those people who may be unaware of their high risk and to encourage them to seek medical care. Since most individuals with undiagnosed diabetes or at high risk are asymptomatic [29], use of simple, safe, and validated tests such as the one used in this project can inform rural West Virginians of their risk and consequences, available programs, and clinics for follow-up care and that the problem is amenable to prevention and control. Furthermore, our results showed that screening was successful for all ages, genders, and minorities and Non-Hispanic Whites (the majority in West Virginia). Yet in order for these community-based screenings to succeed, be sustained, and be effective in West Virginia, it is critical that barriers to care and disparities are also considered. Besides known barriers such as access to care and transportation, other factors that contribute to the diabetes disparities include lack of specialists skilled in diabetic care and a disconnect or distrust between patients and their providers [25, 30]. In prior studies participants perceived that providers did not have a strong understanding of their culture [30] and the need for culturally sensitive programs for West Virginians [31].

The cost of the diabetes screenings was reasonable ($10 per point-of-cost A1c kit) and allowed the project to be clinically, socially, and ethically acceptable for the extension agents. While it can be argued that A1c tests may not be cost-effective for all community-based diabetes screenings, the American Diabetes Association recommends testing to detect type 2 diabetes and prediabetes in asymptomatic adults who are overweight or obese and have one or more additional risk factors for diabetes and in all adults 45 years of age or older [32]. Stepwise screenings [33] can also be used for these community-based diabetes screenings with the first step of the screening to use the 7-item CDC survey followed by A1c tests for those with diabetes risk scores ≥9, however, in a rural state such as West Virginia which had disproportionally higher prevalence rates of obesity (1st) and diabetes (4th) nationally [34], and many people have limited or no access to routine medical care, universal screening using a method that does not require fasting is reasonable. Community-based diabetes screening inevitably involves some concern related to high risk individuals identified by screenings who may not get the care they need and/or follow-up with health care providers for care or additional diagnostic tests. These are valid concerns that bear further exploration and research but do not negate the benefit of this simple screening program that quantifies the risk of undiagnosed diabetes or prediabetes while there is still time to prevent progression and complications.

The low cost of A1c tests could be easily translated for any clinic and hospital and for health care professionals and the public. This the cost of the screening was very reasonable in relation to the expenditure on medical care as a whole and the benefits which far outweighed the time for program planning and implementation. Conducting diabetes screening and referrals also allowed the extension agents for community outreach in their counties to improve diabetes and health outcomes and possibly improve the quality of life. Since the extension agents are trusted members of the community, use of these agents can be a sustainable model for diabetes screenings in community settings.

Using the extension network as a future model for diabetes screenings, referrals and lifestyle programs in West Virginia are innovative for several reasons: (1) extension agents are embedded in the community; they know the leaders and the community resources very well; (2) no other state organization has the outreach or infrastructure; (3) offering community-based screenings falls in direct line with the mission and goals of the land grant university and allows for long-term sustainability of this model; and (4) lessons learned will be shared across state extension programs, providing additional capacity to translate this model widely. In addition, rural communities can also devote resources for these low-cost diabetes screenings to develop public/private and community partnership as the return on investment is high and the condition is amenable to prevention. However, there should be clear guidelines on the management of individuals with a positive test result in order to help to both prevent the disease through lifestyle and pharmacological intervention and reduce the morbidity and mortality associated with diabetes [20].

Results showed a wide gap in reported lifestyle behavior and their overweight and obesity status. For example, approximately two-thirds or 71% of the undiagnosed high risk individuals indicated that they read food labels when making food choices, yet, nearly half of the participants were obese according to their self-reported BMI rankings. This gap between perception and lifestyle behavior has been noted in the literature when individuals who are overweight or obese do not think they are so [11]. While no association existed between educational status and diabetes risk in the logistic regression analysis, more than half of the high risk participants had some college level education or a college degree suggesting that there exists a gap in educated participant's knowledge of risk factors (especially family history of diabetes and hypertension) and the ability to effectively apply that knowledge to lower risk. Furthermore, a lack of perceived personal risk among those with a family history of diabetes and hypertension in this study may be indicative of not applying the knowledge to themselves. This concurs with a national population-based diabetes survey [35] where participants with prediabetes and at risk did not perceive their risk to be high.

Females were at higher risk than males, possibility due to their higher BMI and gestational diabetes. The odds of having higher risk for diabetes was fourfold than males making it critical that public health education should highlight the gender disparity for primary and secondary prevention efforts. This discovery also makes a strong case for continued community-based educational efforts to improve awareness, even among educated individuals to improve preventive efforts such as the adoption of healthy lifestyle in order to reduce the risk of developing type 2 diabetes in rural West Virginia.

There are numerous positive results that were attained in this project. Conducting diabetes screening during health fairs and other public events allowed extension agents to provide greater access to community screening and referrals to health care providers and extension classes such as Dining with Diabetes and strengthened partnerships between community and clinical service agencies to reduce health disparities, improve diabetes and health outcomes, and improve quality of life. Since the extension agents are trusted members of the community, use of these agents can be a sustainable model for diabetes screenings in community settings. However, as future research and screening are conducted on community-based screenings and referrals for treatment, there should be clear guidelines on the management of individuals with a positive test result and consider how to support the autonomy of participants through the lifestyle intervention, diabetes management, and pharmacological intervention in order to help to both prevent the disease and reduce the morbidity and mortality associated with diabetes [20]. The results of the project will be used to develop additional, community-based, culturally competent diabetes prevention and management programs.

Results should be considered in context to the following limitations. One of the limitations of the present study is that a small number of minority adults participated in the screening. This discrepancy poses the threat that the results are only indicative of the non-Hispanic White population and not the population of rural West Virginia, as a whole. However, West Virginia is comprised of a predominantly non-Hispanic White population (97%) and our study represented more minorities than the general population of the state. Studies indicate that ethnicity and minority status are risk factor not only for having type 2 diabetes but also for increasing morbidity and mortality with the disease [36, 37]. Hence, efforts need to be made to improve screening in this group to improve inequalities and compliance as the A1c does not require a fasting blood test. Another potential weakness is that the information was self-reported. Individuals may have answered questions in a socially acceptable manner that would incorporate information bias. For example, reporting that they are physically active incorrectly lowers their risk score for early onset diabetes. Other potential samples include participants that may not be representative of the general West Virginia population as there was a bias towards a higher educated participant group in this study as well as those who may have been motivated to volunteer for the community diabetes screenings due to a greater awareness and/or worry about their health status. A higher risk of diabetes was also noted among females, despite having no gender differences observed in the prevalence of diagnosed diabetes cases in the West Virginia population, according to the Behavioral Risk Factor Surveillance System (BRFSS) data [38]. Hence, future studies may explore the replicability of the high risk among women in larger samples and population-based studies.

## 4. Conclusion

The findings clearly indicated that community based screening was an effective way to assess diabetes risk in rural West Virginia. The findings also indicated that there exists a gap between knowledge of diabetes risk factors and the need for screenings, referrals, and lifestyle programs to effectively lower those risks. Plans for future research include targeting minority groups within the state using similar community outlets and faith-based organizations and enhancing our data collection to include health literacy and more detailed diet and physical activity reports. While individual assessments were beneficial, we feel association of their diabetes risk scores with clinical data (A1c) will be more informative for participants and motivate them to attend* Dining with Diabetes* classes and/or other diabetes education or lifestyle programs available in their communities.

## Figures and Tables

**Table 1:** Sample characteristics of participants by diagnosed diabetes and undiagnosed high risk and low risk (*N* = 538).

Variables	Diagnosed diabetes by a HCP		Undiagnosed; high risk		Low risk		*P* value
	(*n* = 81; 15.2%)		(*n* = 268; 49.8%)		(*n* = 184; 34.2%)		
	Freq.	%	Freq.	%	Freq.	%	
Sex							**0.010**
Female	56	69.1	221	83.7	140	76.1	
Male	25	30.9	43	16.3	44	23.9	
Ethnicity							0.084
Non-Hispanic Whites	77	95.1	238	90.5	158	86.3	
Minorities	4	4.9	25	9.5	25	13.7	
Age	Mean = 51.2 ± 16.4 years						**<0.001**
18–44	13	16.0	40	15.2	134	72.8	
45–64	41	50.6	121	45.8	49	26.6	
≥65	27	33.3	103	39.0	1		
Education							**0.382**
≤High school grad	35	50.7	92	41.3	66	43.4	
College grad or some college	34	49.3	131	58.7	86	56.6	
Exercise at least 30 minutes							**0.481**
Less than once a week	35	43.8	107	40.7	63	34.8	
1-2 times per week	26	32.5	76	28.9	60	33.1	
3-4 or more times per week	19	23.8	80	30.4	58	32.0	
Body mass index	Mean = 31.1 ± 7.5						***<0.001***
Under/normal	5	6.5	38	15.6	55	31.6	
Overweight	22	28.6	85	34.8	53	30.5	
Obese	50	64.9	121	49.6	66	37.9	
Read food labels							***0.008***
Yes	53	66.3	182	70.5	101	56.1	
No	27		76		79		
Smoke							***<0.001***
Yes	11	13.8	21	8.1	42	24.3	
No	69	86.3	239	91.9	131	75.7	
History of hypertension							**0.040**
Yes	64	84.2	180	72.3	119	68.8	
No	12	15.8	69	27.2	54	31.2	
Family history of hypertension							<0.001
Yes	50	64.9	114	46.3	38	22.0	
No	27	35.1	132	53.7	135	78.0	

Note: HCP: healthcare provider.

**Table 2:** Diabetes risk factors by low and high risk for prediabetes.

	Low risk		High risk		
Diabetes risk factors	(*n* = 184)		(*n* = 268)		*P* value
	Freq.	%	Freq.	%	
Baby weigh more than 9 pounds at birth^*∗*^					**0.354**
No	156	88.1	249	86.5	
Yes	21	11.9	39	13.5	
Sister or brother with diabetes					**0.002**
No	153	79.3	210	66.9	
Yes	40	20.7	104	33.1	
Parent with diabetes					**0.106**
No	122	62.9	178	56.9	
Yes	72	37.1	135	43.1	
Weight is more than listed for height					**<0.001**
No	103	56.6	60	19.5	
Yes	79	43.4	247	80.5	
Less than 65 years and get little exercise/day					**<0.001**
No	177	94.7	188	60.1	
Yes	10	5.3	125	39.9	
45–64 years of age					**<0.001**
No	145	71.4	168	51.5	
Yes	58	28.6	158	48.5	
65 years or older					**<0.001**
No	203	100.0	194	59.1	
Yes	—	0.0	134	40.9	

	Mean	SD	Mean	SD	

Mean diabetes risk score	4.15	2.46	12.35	2.78	**<0.001**
Mean A1c	5.6	0.41	5.9	0.85	**<0.001**

Note: low risk for diabetes is defined by risk score < 9; high risk for prediabetes is defined by risk score ≥ 9.

^*∗*^Only females were selected for this analysis.

**Table 3:** Logistic regression: predicting high risk for prediabetes^▽^.

	Unadjusted		Adjusted^*ϕ*^	
	OR	95% CI	OR	95% CI
Sex				
Male			Reference	
Female	1.61^*∗∗*^	1.01, 2.6	3.91^+^	0.89, 17.09
Education				
≤High school grad	0.92	0.60, 1.39	0.73	0.24, 2.19
College grad or some college			Reference	
Physical activity^*∗∗∗*^				
Low	1.23	0.77, 1.95	0.96	0.28, 3.23
Moderate	0.92	0.56, 1.48	0.97	0.28, 3.29
High			Reference	
Siblings with diabetes				
Yes			Reference	
No	0.49^*∗∗*^	0.30, 0.78	0.76	0.22, 2.65
Parents with diabetes				
Yes			Reference	
No	0.69^+^	0.46, 1.04	0.89	0.30, 2.60
Baby weigh more than 9 pounds at birth				
Yes			Reference	
No	1.10	0.59, 2.07	12.2^*∗∗*^	2.6, 56.9
Family history of hypertension				
Yes			Reference	
No	0.85	0.55, 1.29	4.08^*∗*^	1.18, 14.16
Medical history of hypertension				
Yes			Reference	
No	0.32	0.21, 0.50	0.58	0.20, 1.72
Read food labels				
Yes			Reference	
No	0.53^*∗∗*^	0.36, 0.79	0.90	0.33, 2.45
Smoke				
Yes			Reference	
No	3.64^*∗∗*^	2.07, 6.42	0.72	0.21, 2.39
A1c	2.87^*∗∗*^	1.6, 5.2	1.14	0.60, 3.34
Age	1.10^*∗∗*^	1.08, 1.12	1.15^*∗∗*^	1.09, 1.20
BMI	1.05^*∗∗*^	1.02, 1.08	1.23^*∗∗*^	1.12, 1.36

^▽^Individuals with diagnosed diabetes cases by a healthcare professionals were deleted from the analysis.

*P* values: ^+^<0.10, ^*∗*^<0.05, and ^*∗∗*^<0.01.

^*∗∗∗*^Physical activity: low, exercise less than once a week of at least 30 minutes; moderate, exercise 1-2 times per week of at least 30 minutes; high, exercise 3-4 or more times per week of at least 30 minutes.

^*ϕ*^Adjusted ORs reflect the association between high risk prediabetes status and each variable, adjusting for all the other variables in the model.

## References

[1] CDC (2011). *National Diabetes Fact Sheet: National Estimates and General Information on Diabetes and Prediabetes in the United States*.

[2] Resnick H. E., Harris M. I., Brock D. B., Harris T. B. (2000). American Diabetes Association diabetes diagnostic criteria, advancing age, and cardiovascular disease risk profiles: results from the Third National Health and Nutrition Examination Survey. *Diabetes Care*.

[3] CDC (2011). *National Diabetes Fact Sheet*.

[4] Data W. S. (2012). *West Virginia Behavioral Risk Factor Surveillance System Report*.

[5] Institute for Alternative Futures (2010). *Diabetes 2025 Forecasting Model*.

[6] Harris M. I., Eastman R. C. (2000). Early detection of undiagnosed diabetes mellitus: a US perspective. *Diabetes/Metabolism Research and Reviews*.

[7] Boyle J. P., Thompson T. J., Gregg E. W., Barker L. E., Williamson D. F. (2010). Projection of the year 2050 burden of diabetes in the US adult population: dynamic modeling of incidence, mortality, and prediabetes prevalence. *Population Health Metrics*.

[8] Huang E. S., Basu A., O'Grady M., Capretta J. C. (2009). Projecting the future diabetes population size and related costs for the U.S.. *Diabetes Care*.

[9] Tang Y. H., Pang S. M. C., Chan M. F., Yeung G. S. P., Yeung V. T. F. (2008). Health literacy, complication awareness, and diabetic control in patients with type 2 diabetes mellitus. *Journal of Advanced Nursing*.

[10] Rowley K. G., Daniel M., Skinner K., Skinner M., White G. A., O'Dea K. (2000). Effectiveness of a community-directed ‘healthy lifestyle’ program in a remote Australian Aboriginal community. *Australian and New Zealand Journal of Public Health*.

[11] Bupa Health Pulse (2011). *Global Trends, Attitudes, and Influences*.

[12] Ratner R., Goldberg R., Haffner S. (2005). Impact of intensive lifestyle and metformin therapy on cardiovascular disease risk factors in the diabetes prevention program. *Diabetes Care*.

[13] Knowler W. C., Barrett-Connor E., Fowler S. E. (2002). Reduction in the incidence of type 2 diabetes with lifestyle intervention or metformin. *The New England Journal of Medicine*.

[14] Kramer M. K., Kriska A. M., Venditti E. M. (2009). Translating the Diabetes Prevention Program: a comprehensive model for prevention training and program delivery. *American Journal of Preventive Medicine*.

[15] Jackson L. (2009). Translating the diabetes prevention program into practice: a review of community interventions. *Diabetes Educator*.

[16] Balagopal P., Kamalamma N., Patel T. G., Misra R. (2008). A community-based diabetes prevention and management education program in a rural village in India. *Diabetes Care*.

[17] Boltri J. M., Davis-Smith Y. M., Seale J. P., Shellenberger S., Okosun I. S., Cornelius M. E. (2008). Diabetes prevention in a faith-based setting: results of translational research. *Journal of Public Health Management and Practice*.

[18] Ackermann R. T., Finch E. A., Brizendine E., Zhou H., Marrero D. G. (2008). Translating the diabetes prevention program into the community. The DEPLOY Pilot Study. *American Journal of Preventive Medicine*.

[19] Balagopal P., Kamalamma N., Patel T. G., Misra R. (2012). A community-based participatory diabetes prevention and management intervention in rural India using community health workers. *Diabetes Educator*.

[20] Balkau B. (2008). Screening for diabetes. *Diabetes Care*.

[21] ADA (2010). Summary of revisions for the 2010 clinical practice recommendations. *Diabetes Care*.

[22] FDA OTC-Over the Counter Medical Devices. http://www.accessdata.fda.gov/scripts/cdrh/cfdocs/cfIVD/Search.cfm.

[23] Sicard D. A., Taylor J. R. (2005). Comparison of point-of-care HbA_1c_ test versus standardized laboratory testing. *Annals of Pharmacotherapy*.

[24] Manchin J., Curtis C., Bazzle N. M., Slemp C., Toth J. L. (2009). *The Burden of Diabetes in West Virginia*.

[25] Coyne C. A., Demian-Popescu C., Friend D. (2006). Social and cultural factors influencing health in southern West Virginia: a qualitative study. *Preventing Chronic Disease*.

[26] Denham S. A., Wood L. E., Remsberg K. (2010). Diabetes care: provider disparities in the US Appalachian region. *Rural and Remote Health*.

[27] Huttlinger K., Schaller-Ayers J., Lawson T. (2004). Health care in appalachia: a population-based approach. *Public Health Nursing*.

[28] Smith S. L., Tessaro I. A. (2005). Cultural perspectives on diabetes in an Appalachian population. *American Journal of Health Behavior*.

[29] Harris M. I., Klein R., Welborn T. A., Knuiman M. W. (1992). Onset of NIDDM occurs at least 4–7 yr before clinical diagnosis. *Diabetes Care*.

[30] McGarvey E. L., Leon-Verdin M., Killos L. F., Guterbock T., Cohn W. F. (2011). Health disparities between appalachian and non-appalachian counties in Virginia USA. *Journal of Community Health*.

[31] Boltri J. M., Davis-Smith Y. M., Zayas L. E. (2006). Developing a church-based diabetes prevention program with African Americans: focus group findings. *Diabetes Educator*.

[32] American Diabetes Association (2013). Standards of medical care in diabetes—2013. *Diabetes Care*.

[33] Spijkerman A. M. W., Adriaanse M. C., Dekker J. M. (2002). Diabetic patients detected by population-based stepwise screening already have a diabetic cardiovascular risk profile. *Diabetes Care*.

[34] West Virginia Health Statistics Center (2015). *West Virginia Behavioral Risk Factor Surveillance System Report, 2013*.

[35] Piccinino L., Griffey S., Gallivan J., Lotenberg L. D., Tuncer D. (2015). Recent trends in diabetes knowledge, perceptions, and behaviors: implications for national diabetes education. *Health Education & Behavior*.

[36] Sheehy A., Pandhi N., Coursin D. B. (2011). Minority status and diabetes screening in an ambulatory population. *Diabetes Care*.

[37] Meneghini L. (2008). Ethnic disparities in diabetes care: myth or reality?. *Current Opinion in Endocrinology, Diabetes and Obesity*.

[38] West Virginia Health and Human Services HSC Statistical Brief No. 28. Diabetes and Health Equity in West Virginia: A Review. http://www.wvdhhr.org/bph/hsc/pubs/briefs/028/brief28_20121220_health_eq_stat.pdf.

